# Temporal shifts in public perceptions of influenza and its vaccine before, during, and after COVID-19 in a fragile nation

**DOI:** 10.1080/07853890.2026.2708521

**Published:** 2026-08-20

**Authors:** Wesam S. Ahmed, Abdulsalam M. Halboup, Hamada R. H. Al-Absi, Sayida Al-Jamei, Ahmed Al-mohamadi, Arwa Alshargabi, Yousf K. Al-Ashbat, Karem H. Alzoubi

**Affiliations:** a College of Health and Life Sciences, Hamad Bin Khalifa University, Doha, Qatar; b Department of Clinical Pharmacy and Pharmacy Practice, Faculty of Pharmacy, University of Science and Technology, Sana’a, Yemen; c Department of Clinical Pharmacy, School of Pharmaceutical Sciences, University Sains Malaysia, Penang, Malaysia; d Department of Computer and Software Engineering, Barzan University College, Doha, Qatar; e Department of Pharmacy, Faculty of Health Sciences, Yemen University, Sana’a, Yemen; f Pharmacy Department, Faculty of Medical Sciences, Saba University, Sana’a, Yemen; g Department of Clinical Pharmacy and Therapeutics, Faculty of Pharmacy, Al-Razi University, Sana’a, Yemen; h Department of Pharmaceutical Sciences, College of Pharmacy, QU Health, Qatar University, Doha, Qatar

**Keywords:** COVID-19, influenza, awareness, vaccine, fragile nation, pandemic, perceptions, Yemen, conflict, LMIC

## Abstract

**Background:**

Since the emergence of the COVID-19 pandemic, co-infection with COVID-19 and influenza has been associated with adverse health and economic outcomes. Emerging evidence suggests that the COVID-19 pandemic has influenced public perception of infections, including influenza, and acceptance of its vaccine.

**Objectives:**

To assess temporal shifts in public knowledge of influenza and attitudes toward its vaccine before (Phase-I), during (Phase-II), and after (Phase-III) the COVID-19 pandemic in Yemen, a low-income, fragile nation.

**Methods:**

A survey questionnaire was distributed to the public in Yemen, and a total of 1,856 participants completed the survey (Phase-I = 433, Phase-II = 981, Phase-III = 442).

**Results:**

There was an overall increase in influenza awareness in Phases-II and III compared to Phase-I. This rise was paralleled by a change in the source from which participants gathered information about influenza. Although awareness increased, a decline in future influenza vaccine acceptance was observed. However, participants in Phases II and III showed greater willingness to receive the vaccine if it was endorsed by the government and doctors, confirmed to be safe and effective, and provided at no cost by the authorities. Logistic regression and machine-learning models highlighted predictors of high influenza awareness and future vaccine acceptance among participants in each phase.

**Conclusion:**

The findings illustrate vaccine behaviour dynamics in a fragile country across the pandemic timeline and provide actionable insights for health authorities for designing effective vaccination campaigns and implementing vaccination policies tailored to the unique challenges faced during pandemics and health crises.

## Introduction

The coronavirus disease 2019 (COVID-19) pandemic ranks among the most significant global health crises in modern history, reshaping public health priorities and individual health behaviours in unprecedented ways [1–3]. While efforts to contain the pandemic dominated healthcare systems worldwide, the interaction between COVID-19 infodemics and health literacy emerged as a critical area of study [4–6]. COVID-19 also shares clinical symptoms with other respiratory infections, most notably influenza, with coinfection associated with poor outcomes [7–9]. The influenza vaccine is a key intervention to prevent influenza and its complications [10], and has been linked to lower SARS-CoV-2 seroprevalence, reduced hospital and intensive care unit admissions, and decreased mortality from COVID-19 [11]. Consequently, protection against influenza has gained increased importance during the COVID-19 pandemic. Additionally, recent studies suggest that perceived vulnerability to COVID-19 is a positive predictor of influenza vaccine uptake [12,13].

However, the impact of infodemics–that is, the overabundance of information, both accurate and inaccurate–and low health literacy in conflict-affected settings like Yemen remains largely unexplored. Yemen has endured a decade-long armed conflict that has severely weakened its infrastructure, already strained by political instability, famine, human displacement, and war-related injuries. The country faces a compounded burden of neglected tropical diseases [14], exacerbated by the COVID-19 pandemic [15], which has further overwhelmed its healthcare system. Recent reports also suggest a rise in influenza incidence [16], placing additional pressure on already limited resources.

Compounding these challenges, the influenza vaccine remained excluded from Yemen’s national immunization program before, during, and after the COVID-19 pandemic [17,18]. Consequently, public perceptions and attitudes toward vaccination with respect to the pandemic timeline may influence morbidity, mortality, and overall burden on the healthcare system during pandemics. In our previous analysis [17], data from participants surveyed before and during the COVID-19 pandemic were analyzed collectively to provide an overall picture of public perceptions of influenza and its vaccine. Emerging evidence highlighted the pandemic’s potential impact on vaccine awareness and acceptance [19,20]. To address this potential temporal effect, we conducted an additional recruitment phase following the pandemic’s subsidence and examined the data by period. Accordingly, the present study analyses all three recruitment phases separately (before, during, and after the COVID-19 pandemic) to assess temporal changes in public knowledge of influenza and attitudes toward its vaccine. Furthermore, we incorporated machine-learning models, in addition to the traditional regression analysis, to identify factors associated with high influenza awareness and vaccine acceptance in each time period. The study presents new insights into the temporal dynamics of vaccine awareness and acceptance, offering actionable implications for improving vaccine uptake in fragile and conflict-affected settings during health crises.

## Methods

### Research design and participant characteristics

This cross-sectional observational study builds on our previously published pooled analysis, which assessed general public awareness and attitudes toward influenza vaccination without reference to the COVID-19 timeline [17]. The current work adds a new post-pandemic recruitment phase and reanalyses all data from a temporal perspective. Data from all three phases were analysed separately in the present study to examine temporal differences in influenza-related knowledge and vaccine attitudes. A machine learning approach was introduced to assess predictors of high influenza awareness and vaccine acceptance across different phases. Participants in all phases were recruited using the same inclusion criteria and completed the same validated questionnaire. Phase-I (pre-pandemic, March 2019–February 2020) involved face-to-face interviews at public venues in Sana’a. Due to the COVID-19 outbreak, Phase-II (during-pandemic, July–October 2020) utilized an online format, with the survey distributed *via* social media platforms (WhatsApp and Facebook), reaching participants nationwide. The final phase, Phase-III (post-pandemic, December 2023), was also conducted online one year after global COVID-19 restrictions were lifted. The survey targeted individuals in Yemen who were 18 years or older, autonomous, and capable of reading and understanding Arabic. During the in-person interviews, participants provided oral consent to participate. For those recruited online, a consent statement was placed at the start of the survey and included in the enrollment invitation disseminated on social media outlets. The study was approved by the Ethics Committee without a written consent requirement. The study was conducted in accordance with the principles outlined in the Declaration of Helsinki.

### Survey questionnaire

The survey was validated previously [21] and administered in Arabic. The same questionnaire was used in the three phases of data collection to ensure comparability across phases. The questionnaire included various response formats, such as multiple-choice, ‘Yes,’ ‘No,’ ‘I am not sure,’ multiple checkboxes, Likert scale items, and open-ended questions. For the purpose of analysis, the survey was divided into four sections: (1) Demographic information; (2) Awareness of influenza, which assessed participants’ understanding of influenza, including its transmission modes and preventive measures; (3) Source of information, which identified where participants obtained their information about influenza and its vaccine; and (4) Attitudes toward the influenza vaccine, which explored participants’ willingness to receive the vaccine and the motivating factors for the latter.

### Statistical analysis

The survey data were imported into IBM SPSS Statistics, version 27.0. All percentages were rounded to one decimal place in tables and figures, and to whole numbers in the text. Chi-square tests were used to examine the association between the phase of COVID-19 (Phase-I, Phase-II, or Phase-III) and participants’ awareness of influenza. Awareness levels were classified into two groups: high awareness (score ≥ 80%) and low awareness (score < 80%) [22]. The five-point Likert scale that was originally applied to the five motivation-related questions was simplified to a three-point format: ‘strongly agree/agree’, ‘neutral’, and ‘strongly disagree/disagree’, where lower scores indicated greater motivation. Participants’ motivation was categorized into two groups: ‘generally motivated’ (scores ≤ 25th percentile) and ‘generally reserved’ (scores > 25th percentile) [18] using the 25th percentile as the threshold.

Identification of variables associated with high influenza awareness and future influenza vaccine acceptance was carried out using univariable and multivariable logistic regression within a univariate framework. Variables with a *p*-value less than 0.25 in the univariable logistic regression were included in the multivariable model. Crude odds ratios (CORs) and adjusted odds ratios (AORs) were reported for the univariable and multivariable analyses, respectively. For all analyses, the 95% confidence intervals (CIs) and *p*-values were reported. A *p*-value of less than 0.05 was considered statistically significant.

The random forest (RF) machine-learning model was used to rank the predictors’ contribution to the participants’ high influenza awareness and their acceptance of the vaccine in each phase of the COVID-19 pandemic. RF works by building multiple decision trees, each one giving a prediction. The algorithm then combines those predictions to make a final decision. The model is good at handling complex data and provides insights into the contribution of each feature (factor) to the prediction model’s performance [23], which can be used to find covariates having the highest influence on the prediction outcome [24,25]. The algorithm is commonly used for classification or regression tasks. For classification tasks, as in both cases here, RF selects the class of the majority vote from the trees. In evaluating the performance of the RF model for the prediction tasks, we used the Receiver Operating Characteristic – Area Under the Curve (ROC-AUC) value as the main performance metric. The ROC-AUC metric provides a comprehensive measure of the model’s distinguishing ability between classes. To find the best parameters for the RF model that maximize the ROC-AUC score, and to ensure the model was neither overfitting nor underfitting, we tuned the RF model using GridSearch to optimize its performance. This was done by exploring various combinations of hyperparameters such as the number of trees, maximum depth, and minimum sample split. The hyperparameters were tuned as follows: number of trees = 100, maximum depth = 8, maximum features = ‘sqrt’, minimum sample leaf = 2, and minimum sample split = 5. All reported results are presented in the form of tables or figures.

## Results

### Demographic characteristics of participants

Demographic data of the participants (*n* = 1856) revealed notable trends across the different phases of the COVID-19 pandemic (Table 1). The majority of participants were aged 25 years or younger (*n* = 961, 52%), although Phase-II had a higher proportion of participants older than 25 years (*n* = 567, 58%). The gender distribution was predominantly male (*n* = 1147, 62%), with the highest male participation occurring in Phase-II (*n* = 627, 64%). Representation by governorate varied, with Sana’a showing the highest participation (*n* = 668, 36%), particularly in Phases-II and III. Administratively, Yemen comprises 21 governorates and the capital municipality of Sana’a (Amanat Al Asimah), which functions as a separate first-level administrative unit rather than being part of Sana’a Governorate. In everyday usage, however, Yemenis often refer to both Sana’a City and Sana’a Governorate simply as “Sana’a,” and the distinction is not always made outside formal administrative contexts. Therefore, Sana’a, in the context of this paper, includes both Sana’a Governorate and the capital municipality of Sana’a. In terms of marital status, most participants were single (*n* = 1040, 56%), though a larger proportion of married participants was observed in Phase-II (*n* = 491, 50%). Regarding education, the majority held a bachelor’s degree (*n* = 1166, 63%), with a notable increase in postgraduate degree holders during Phase-II (*n* = 123, 13%). Employment status also varied, with a higher proportion of part-time (*n* = 185, 18.9%) and full-time employment (*n* = 315, 32%) reported in Phase-II. A significant number of participants were employed in the medical field, particularly in Phase-III (*n* = 259, 59%). Concerning family and health-related demographics, most participants did not have children (*n* = 1,326, 71%) or medical insurance (*n* = 1,451, 78%). Smoking was relatively uncommon (*n* = 205, 11%), and the majority reported no chronic diseases (*n* = 1,612, 87%). Pregnancy was rare among female participants (*n* = 27, 4%). A detailed overview of the participants’ demographic characteristics across the different COVID-19 phases is provided in Table 1.

**Table 1.** Demographic data of participants (*n* = 1856).

Parameter		COVID-19 Phase^a^			Total
		Phase-I	Phase-II	Phase-III	
		n (%)	n (%)	n (%)	n (%)
Age, median (IQR)		24 (8)	30 (14)	24 (8)	25 (11)
Gender					
	Male	258 (59.6)	627 (63.9)	262 (59.3)	1147 (61.8)
	Female	175 (40.4)	354 (36.1)	180 (40.7)	709 (38.2)
Governorate					
	Sana’a	125 (28.9)	355 (36.2)	188 (42.5)	668 (36.0)
	Taiz	108 (24.9)	221 (22.5)	118 (26.7)	447 (24.1)
	Ibb	63 (14.5)	114 (11.6)	43 (9.7)	220 (11.9)
	Other cities	137 (31.6)	291 (29.7)	93 (21.0)	521 (28.1)
Marital status					
	Married	139 (32.1)	491 (50.1)	151 (34.2)	781 (42.1)
	Widowed/Divorced	8 (1.8)	22 (2.2)	5 (1.1)	35 (1.9)
	Single	286 (66.1)	468 (47.7)	286 (64.7)	1040 (56.0)
Education level					
	Secondary school	142 (32.8)	133 (13.6)	79 (17.9)	354 (19.1)
	Diploma	38 (8.8)	84 (8.6)	34 (7.7)	156 (8.4)
	Bachelor	240 (55.4)	641 (65.3)	285 (64.5)	1166 (62.8)
	Postgraduate	13 (3.0)	123 (12.5)	44 (10.0)	180 (9.7)
Employment					
	None	293 (67.7)	481 (49.0)	263 (59.5)	1037 (55.9)
	Part-time	70 (16.2)	185 (18.9)	77 (17.4)	332 (17.9)
	Full-time	70 (16.2)	315 (32.1)	102 (23.1)	487 (26.2)
Working in the medical field					
	Yes	123 (28.4)	391 (39.9)	259 (58.6)	773 (41.6)
	No	310 (71.6)	590 (60.1)	183 (41.4)	1083 (58.4)
Having children ≤ 5 years old					
	Yes	82 (18.9)	325 (33.1)	123 (27.8)	530 (28.6)
	No	351 (81.1)	656 (66.9)	319 (72.2)	1326 (71.4)
If children under 5, specify age.					
	< 6 months	8 (9.8)	28 (8.6)	6 (4.9)	42 (7.9)
	6 months–2 years	18 (22.0)	99 (30.5)	48 (39.0)	165 (31.1)
	3–5 years	56 (68.3)	198 (60.9)	69 (56.1)	323 (60.9)
Having medical insurance					
	Yes	96 (22.2)	244 (24.9)	65 (14.7)	405 (21.8)
	No	337 (77.8)	737 (75.1)	377 (85.3)	1451 (78.2)
Smoking status					
	Yes	59 (13.6)	113 (11.5)	33 (7.5)	205 (11.0)
	No	374 (86.4)	868 (88.5)	409 (92.5)	1651 (89.0)
Having one or more chronic diseases					
	Yes	44 (10.2)	128 (13.0)	72 (16.3)	244 (13.1)
	No	389 (89.8)	853 (87.0)	370 (83.7)	1612 (86.9)
Pregnancy status					
	Yes	12 (6.9)	13 (3.8)	2 (1.1)	27 (3.9)
	No	162 (93.1)	331 (96.2)	175 (98.9)	668 (96.1)

^a^ Reported percentages are based on the total number of participants in each phase, except for the child-age and pregnancy-status categories, which are based on the relevant subgroup denominators.

### Awareness of influenza during different phases of the COVID-19 pandemic

Table 2 presents respondents’ awareness of influenza across different phases of the COVID-19 pandemic, highlighting significant improvements over time. In Phase-I, 74% (*n* = 322) of participants recognized influenza as a respiratory infection that can lead to severe pneumonia, compared to 86% (*n* = 848) in Phase-II and 90% (*n* = 397) in Phase-III (*p* < 0.001). Similarly, the awareness that “swine flu is a type of influenza” increased significantly from 45% (*n* = 193) in Phase-I to 75% (*n* = 735) in Phase-II, and 59% (*n* = 262) in Phase-III (*p* < 0.001). Awareness of preventive measures also improved. For instance, the belief that influenza spreads through respiratory droplets increased from 77% (*n* = 334) in Phase-I to 86% (*n* = 846) in Phase-II and 90% (*n* = 397) in Phase-III (*p* < 0.001). The awareness that wearing masks can prevent the spread of influenza rose from 88% (*n* = 380) in Phase-I to 96% (*n* = 423) in Phase-III (*p* < 0.001). Additionally, understanding that vaccination is the most effective preventive measure improved from 60% (*n* = 258) in Phase-I to 73% (*n* = 321) in Phase-III (*p* < 0.001). The awareness level significantly improved as well, with the proportion of participants demonstrating high awareness (≥80%) increasing from 22% (*n* = 96) in Phase-I to 51% (*n* = 499) in Phase-II, with a slight decrease to 48% (*n* = 214) in Phase-III (*p* < 0.001).

**Table 2.** Respondents’ awareness of influenza during different phases of the COVID-19 pandemic (*n* = 1856).

Statement		Correct Answers^a^			Chi-square	*P* value
		Phase-I, *n* (%)	Phase-II, *n* (%)	Phase-III, *n* (%)		
Influenza is a contagious respiratory disease caused by viruses like H1N1, which can result in mild to severe illness‡		322 (74.4)	848 (86.4)	397 (89.8)	46.153	<0.001*
Swine flu is caused by the H1N1 virus‡		193 (44.6)	735 (74.9)	262 (59.3)	126.185	<0.001*
All cases of H1N1 require hospitalization§		35 (8.1)	269 (27.4)	105 (23.8)	69.920	<0.001*
All H1N1 infections result in death§		169 (39.0)	592 (60.3)	210 (47.5)	60.089	<0.001*
There is a cure for severe complications caused by influenza§		70 (16.2)	166 (16.9)	61 (13.8)	2.219	0.330
Individuals with chronic conditions are at a higher risk of flu complications‡		274 (63.3)	800 (81.5)	378 (85.5)	76.977	<0.001*
Elderly and young children are at higher risk for flu complications‡		311 (71.8)	695 (70.8)	350 (79.2)	11.205	0.004*
Flu can spread through respiratory droplets from infected individuals‡		334 (77.1)	846 (86.2)	397 (89.8)	30.177	<0.001*
Coughing, sneezing, or talking can spread flu droplets‡		375 (86.6)	903 (92.0)	422 (95.5)	22.910	<0.001*
Touching your mouth or nose after contact with contaminated surfaces can spread flu‡		318 (73.4)	788 (80.3)	336 (76.0)	8.841	0.012*
Wearing a mask can reduce flu transmission‡		380 (87.8)	861 (87.8)	423 (95.7)	23.188	<0.001*
Covering your mouth when sneezing helps reduce flu transmission‡		369 (85.2)	899 (91.6)	424 (95.9)	31.724	<0.001*
Washing hands after coughing or sneezing helps prevent flu spread‡		362 (83.6)	904 (92.2)	423 (95.7)	42.454	<0.001*
Avoiding crowded areas helps reduce flu transmission‡		362 (83.6)	915 (93.3)	421 (95.2)	46.605	<0.001*
Getting vaccinated reduces flu spread‡		258 (59.6)	627 (63.9)	321 (72.6)	17.849	<0.001*
Awareness level	low awareness (Score < 80%)	337 (77.8)	482 (49.1)	228 (51.6)	107.88	<0.001*
	high awareness (Score ≥ 80%)	96 (22.2)	499 (50.9)	214 (48.4)		

^a^ Reported percentages are based on the total number of participants in each phase.

Asterisk and bold text indicate significant association.

^‡^ The correct response to these items is “Yes”.

^§^ The correct response to these items is “No”.

### Source of information

**Figure 1.**
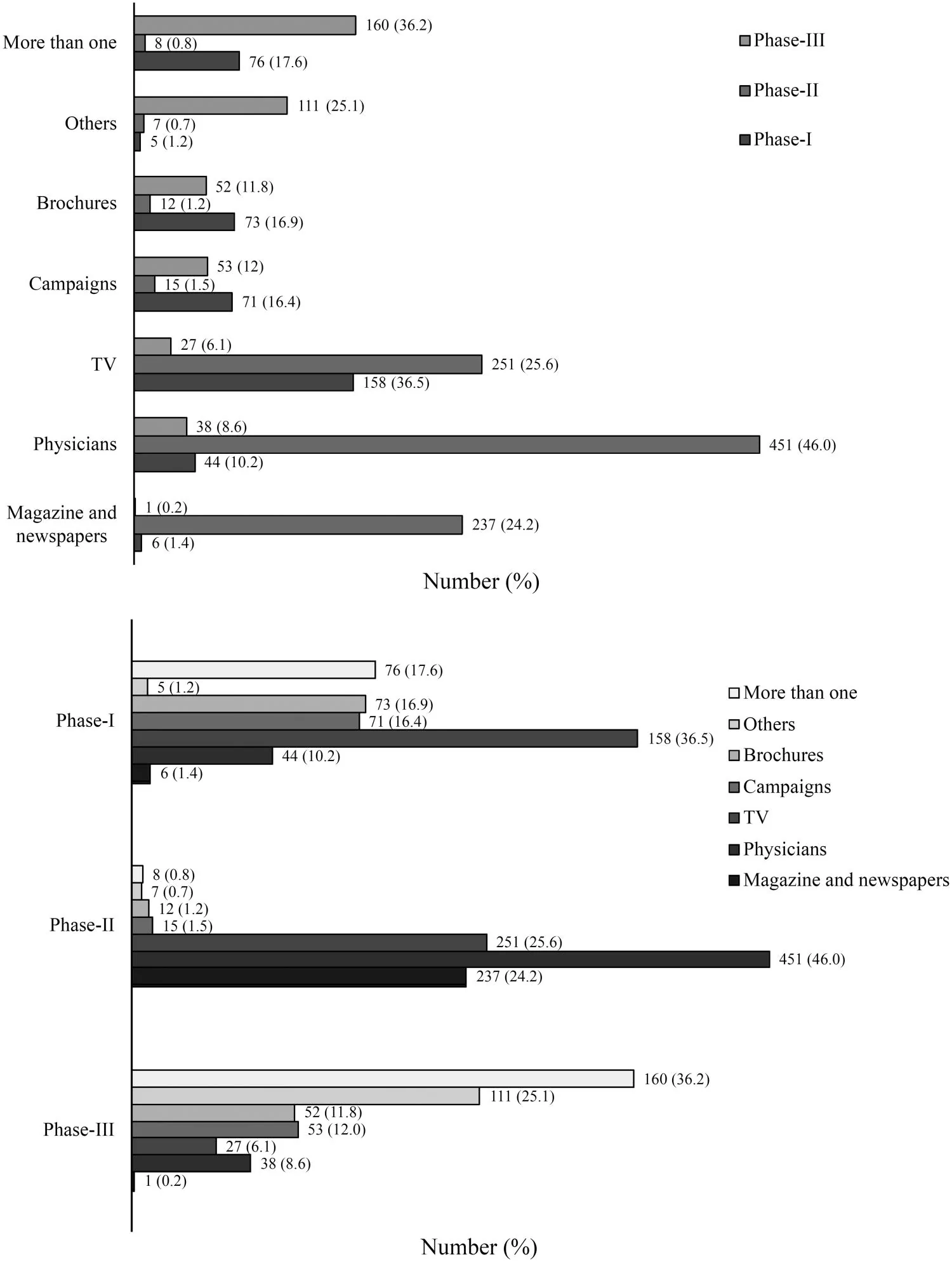
Sources of influenza information utilized by participants during different phases of the COVID-19 pandemic. Upper panel: bar graphs arranged by source of information. Lower panel: bar graphs arranged by Phase. Total participants: 1856 (Phase-I: 433, Phase-II: 981, Phase-III: 442). Numbers in parentheses indicate the percentage out of the total in each phase.

In Phase-I, TV (*n* = 158, 37%) and physicians (*n* = 44, 10%) were the primary sources of information about influenza. In Phase-II, physicians (*n* = 451, 46%) became the predominant source, followed by TV (*n* = 251, 26%) and newspapers (*n* = 237, 24%). During Phase-III, multiple sources (*n* = 160, 36%) and other undetermined sources (*n* = 111, 25%) were the most utilized (Figure 1).

### Factors influencing awareness of influenza

Table 3 presents factors influencing influenza awareness identified through univariable and multivariable logistic regression models. In the univariable analysis, several factors showed significant associations with high awareness: being single (COR = 0.77, 95% CI 0.64–0.93, *p* = 0.006) and smoker (COR = 0.60, 95% CI 0.44–0.81, *p* = 0.001) were associated with lower odds, while higher education levels such as diploma (COR = 1.63, 95% CI 1.10–2.42, *p* = 0.016), bachelor’s degree (COR = 2.20, 95% CI 1.70–2.85, *p* < 0.001), and postgraduate degree (COR = 3.64, 95% CI 2.50–5.30, *p* < 0.001) had higher odds. Employment was also positively associated with high awareness, whether it is part-time (COR = 1.38, 95% CI 1.07–1.77, *p* = 0.012) or full-time employment (COR = 1.39, 95% CI 1.12–1.72, *p* = 0.003). Using physicians as a reference for the source of information, we found that newspapers/magazines were associated with high awareness (COR = 2.06, 95% CI 1.51–2.82, *p* < 0.001), while TV (COR = 0.55, 95% CI 0.42–0.71, *p* < 0.001) and campaigns (COR = 0.33, 95% CI 0.21–0.51, *p* < 0.001) were both negatively associated with high influenza awareness. Additionally, working in the medical field (COR = 2.72, 95% CI 2.25–3.29, *p* < 0.001), and being surveyed during (COR = 3.68, 95% CI 2.84–4.78, *p* < 0.001) or after (COR = 3.34, 95% CI 2.49–4.48, *p* < 0.001) the COVID-19 outbreak were all associated with greater odds of having higher influenza awareness.

**Table 3.** Factors influencing high influenza awareness (*n* = 1856).

Factor		Univariable logistic regression		Multivariable logistic regression	
		COR (95% CI)	*P* Value	AOR (95% CI)	*P* value^a^
Gender	Male	Reference		Reference	
	Female	0.90 (0.74, 1.08)	0.261		
Age		1.01 (1.00, 1.03)	0.010*	1.00 (0.98, 1.02)	0.857
Governorate	Sana’a	Reference			
	Taiz	0.99 (0.780, 1.27)	0.958		
	Ibb	1.08 (0.79, 1.47)	0.625		
	Other cities	1.03 (0.82, 1.30)	0.772		
Marital status	Married	Reference		Reference	
	Widowed/Divorced	1.34 (0.68, 2.64)	0.399	1.85 (0.88, 3.88)	0.105
	Single	0.77 (0.64, 0.93)	0.006*	0.70 (0.52, 0.96)	0.025*
Education level	Secondary school or less	Reference		Reference	
	Diploma	1.63 (1.10, 2.42)	0.016*	1.01 (0.65, 1.56)	0.970
	Bachelor	2.20 (1.70, 2.85)	<0.001*	1.30 (0.97, 1.74)	0.082
	Postgraduate	3.64 (2.50, 5.30)	<0.001*	1.61 (1.03, 2.52)	0.035*
Employment	None	Reference		Reference	
	Part-time	1.38 (1.07, 1.77)	0.012*	1.00 (0.75, 1.33)	0.998
	Full-time	1.39 (1.12, 1.72)	0.003*	1.01 (0.76, 1.33)	0.955
Working in the medical field	No	Reference		Reference	
	Yes	2.72 (2.25, 3.29)	<0.001*	2.60 (2.08, 3.25)	<0.001*
Having children ≤ 5 years old	No	Reference		Reference	
	Yes	1.20 (0.98, 1.47)	0.074	0.87 (0.66, 1.15)	0.330
Having medical insurance	No	Reference		Reference	
	Yes	1.19 (0.96, 1.49)	0.121	1.23 (0.95, 1.58)	0.114
Smoking status	No	Reference		Reference	
	Yes	0.60 (0.44, 0.81)	0.001*	0.60 (0.43, 0.83)	0.003*
Having chronic diseases?	No	Reference			
	Yes	0.94 (0.72, 1.23)	0.655		
Source of Information	Physicians	Reference			
	Newspapers and Magazines	2.06 (1.51, 2.82)	<0.001*	1.66 (1.20, 2.31)	0.002*
	TV	0.55 (0.42, 0.71)	<0.001*	0.76 (0.57, 1.02)	0.066
	Campaigns	0.33 (0.21, 0.51)	<0.001*	0.63 (0.38, 1.05)	0.076
	Brochures	0.75 (0.51, 1.10)	0.137	1.31 (0.82, 2.11)	0.264
	Others	1.17 (0.79, 1.73)	0.445	1.53 (0.92, 2.56)	0.101
	More than one	1.29 (0.95, 1.74)	0.105	2.14 (1.40, 3.25)	<0.001*
Phase of COVID-19	Phase-I	Reference		Reference	
	Phase-II	3.68 (2.84, 4.78)	<0.001*	3.21 (2.28, 4.54)	<0.001*
	Phase-III	3.34 (2.49, 4.48)	<0.001*	1.93 (1.37, 2.73)	<0.001*

^a^ Variables with a p-value of less than 0.25 in the univariable analysis were included in the multivariable logistic regression. A p-value of less than 0.05 was considered statistically significant.

Asterisk and bold text indicate significant association.

Abbreviations: COR, crude odd ratio; AOR, adjusted odd ratio; CI, confidence interval.

When adjusting for confounders in the multivariable model, higher education remained a significant predictor of high awareness. Holding a postgraduate degree (AOR = 1.61, 95% CI 1.03–2.52, *p* = 0.035) was positively associated with high awareness of influenza. Working in the medical field was also strongly associated with high awareness (AOR = 2.60, 95% CI 2.08–3.25, *p* < 0.001). Compared with physicians, obtaining information from newspapers/magazines (AOR = 1.66, 95% CI 1.20–2.31, *p* = 0.002) and from more than one source (AOR = 2.14, 95% CI 1.40–3.25, *p* < 0.001) remained independently associated with higher awareness. Additionally, the survey timing during (AOR = 3.21, 95% CI 2.28–4.54, *p* < 0.001) or after (AOR = 1.93, 95% CI 1.37–2.73, *p* < 0.001) the pandemic remained a significant predictor of high awareness in the multivariable model. Conversely, smoking (AOR = 0.60, 95% CI 0.43–0.83, *p* = 0.003) and being single (AOR = 0.70, 95% CI 0.52–0.96, *p* = 0.025) were both associated with lower odds of having high awareness. Other variables that were significant in the univariable model, such as age and employment, did not show a significant association in the multivariable analysis.

### Future acceptance of the influenza vaccine

**Figure 2.**
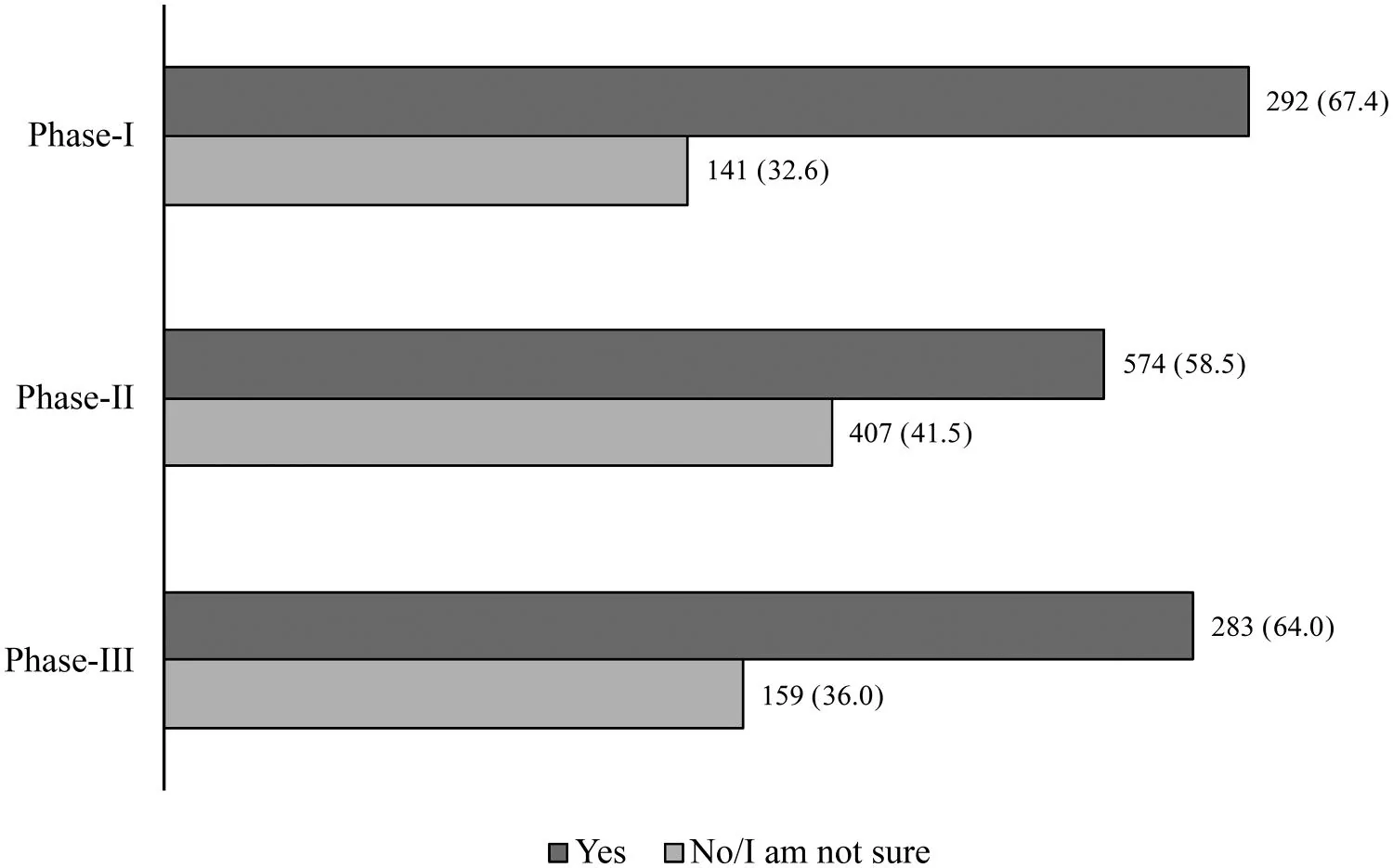
Respondents’ willingness to get vaccinated against influenza in the future (*n* = 1856). n (%): numbers in ­parentheses indicate the percentage out of the total in each phase.

Before the pandemic (Phase-I), 67% (*n* = 292) of participants expressed willingness, compared to 59% (*n* = 574) during the pandemic (Phase-II), and 64% (*n* = 283) after the pandemic (Phase-III) (Figure 2).

### Motivating factors for influenza vaccine acceptance

Table 4 outlines the motivating factors influencing future vaccination intentions reported during various phases of the COVID-19 outbreak. During Phase-I, 54% (*n* = 234) of respondents were motivated by government endorsement of the vaccine. This number increased to 68% (*n* = 670) in Phase-II and 72% (*n* = 318) in Phase-III. More respondents in Phase-II (*n* = 703, 72%) and Phase-III (*n* = 344, 78%) than in Phase-I (*n* = 237, 55%) were motivated to accept the vaccine if it underwent further validation for safety and efficacy. Influenza vaccine endorsement by a physician motivated 61% (*n* = 265) in Phase-I, which is less than those motivated in Phase-II (*n* = 691, 70%) and Phase-III (*n* = 352, 80%). Offering the influenza vaccine free of charge by the government was a motivator for 47% (*n* = 203) of participants in Phase-I, 65% (*n* = 641) in Phase-II, and 67% (*n* = 297) in Phase-III. Overall, more participants were generally motivated in Phase-II (*n* = 480, 49%) and Phase-III (*n* = 232, 53%) compared to Phase-I (*n* = 134, 31%).

**Table 4.** Motivating factors for influenza vaccine acceptance (*n* = 1856).

Factor		Phase of COVID-19^a^					
		Phase-I		Phase-II		Phase-III	
		Count	%	Count	%	Count	%
Vaccine uptake is encouraged by the government	Strongly agree/agree	234	54.0	670	68.3	318	71.9
	Not sure	88	20.3	180	18.3	82	18.6
	Strongly disagree/disagree	111	25.6	131	13.4	42	9.5
The vaccine is more validated for safety and efficacy	Strongly agree/agree	237	54.7	703	71.7	344	77.8
	Not sure	131	30.3	201	20.5	75	17.0
	Strongly disagree/disagree	65	15.0	77	7.8	23	5.2
The vaccine is recommended by the physician	Strongly agree/agree	265	61.2	691	70.4	352	79.6
	Not sure	110	25.4	205	20.9	69	15.6
	Strongly disagree/disagree	58	13.4	85	8.7	21	4.8
The vaccine is offered free of charge by the government	Strongly agree/agree	203	46.9	641	65.3	297	67.2
	Not sure	131	30.3	199	20.3	89	20.1
	Strongly disagree/disagree	99	22.9	141	14.4	56	12.7
Motivation inclination	Generally motivated	134	30.9	480	48.9	232	52.5
	Generally reserved	299	69.1	501	51.1	210	47.5

^a^ Reported percentages are based on the total number of participants in each phase.

### Factors influencing influenza vaccine acceptance

Table 5 shows factors influencing future vaccination willingness among 1856 participants. In univariable analysis, female gender (COR = 0.57, 95% CI: 0.47–0.70, *p* < 0.001), increasing age (COR = 0.98, 95% CI: 0.97–0.99, *p* < 0.001), and postgraduate education (COR = 0.45, 95% CI: 0.31–0.65, *p* < 0.001) were all associated with decreased willingness. Conversely, participants with chronic diseases (COR = 1.49, 95% CI: 1.11–1.99, *p* = 0.008) and those who are generally motivated to receive the vaccine (COR = 2.60, 95% CI: 2.13–3.16, *p* < 0.001) showed higher acceptance. Phase-II was associated with vaccine hesitancy (COR = 0.68, 95% CI: 0.54–0.86, *p* = 0.002), and full-time employment as well (COR = 0.77, 95% CI: 0.62–0.96, *p* = 0.020).

**Table 5.** Factors influencing acceptance of the influenza vaccine (*n* = 1856).

Factor		Univariable logistic regression		Multivariable logistic regression	
		COR (95% CI)	*P* value	AOR (95% CI)	*P* value^a^
Gender	Male	Reference		Reference	
	Female	0.57 (0.47, 0.70)	<0.001*	0.61 (0.49, 0.76)	<0.001*
Age		0.98 (0.97, 0.99)	<0.001*	0.98 (0.97, 1.001)	0.064
Governorate	Sana’a	Reference		Reference	
	Taiz	1.43 (1.12, 1.83)	0.004*	1.25 (0.96, 1.63)	0.091
	Ibb	1.65 (1.20, 2.27)	0.002*	1.39 (0.99, 1.95)	0.058
	Other cities	1.69 (1.33, 2.15)	<0.001*	1.48 (1.14, 1.91)	0.003*
Marital status	Married	Reference		Reference	
	Widowed/Divorced	0.52 (0.26, 1.03)	0.059	0.49 (0.24, 1.03)	0.058
	Single	1.03 (0.85, 1.25)	0.775	0.82 (0.63, 1.06)	0.127
Education level	Secondary school or less	Reference		Reference	
	Diploma	0.88 (0.59, 1.31)	0.541	0.84 (0.55, 1.29)	0.430
	Bachelor	0.78 (0.61, 1.01)	0.056	0.78 (0.59, 1.04)	0.096
	Postgraduate	0.45 (0.31, 0.65)	<0.001*	0.48 (0.31, 0.74)	0.001*
Employment	None	Reference		Reference	
	Part-time	0.87 (0.67, 1.12)	0.280	1.09 (0.81, 1.45)	0.576
	Full-time	0.77 (0.62, 0.96)	0.020*	0.90 (0.68, 1.19)	0.472
Working in the medical field	No	Reference		Reference	
	Yes	1.17 (0.97, 1.41)	0.111	1.05 (0.84, 1.31)	0.673
Having children ≤ 5 years old	No	Reference		Reference	
	Yes	1.02 (0.83, 1.26)	0.841		
Having medical insurance	No	Reference		Reference	
	Yes	0.81 (0.65, 1.02)	0.069	0.82 (0.64, 1.05)	0.118
Smoking status	No	Reference		Reference	
	Yes	1.21 (0.89, 1.64)	0.218	1.09 (0.78, 1.52)	0.618
Having any chronic diseases	No	Reference		Reference	
	Yes	1.49 (1.11, 1.99)	0.008*	1.70 (1.23, 2.33)	0.001*
Motivation inclination	Generally reserved	Reference		Reference	
	Generally motivated	2.60 (2.13, 3.16)	<0.001*	2.82 (2.29, 3.47)	<0.001*
Phase of COVID-19	Phase-I	Reference		Reference	
	Phase-II	0.68 (0.54, 0.86)	0.002*	0.62 (0.48, 0.81)	<0.001*
	Phase-III	0.86 (0.65, 1.14)	0.288	0.75 (0.55, 1.02)	0.071

^a^ Variables with a p-value of less than 0.25 in the univariable analysis were included in the multivariable logistic regression. A p-value of less than 0.05 was considered statistically significant.

Asterisk and bold text indicate significant association.

Abbreviations: COR, crude odd ratio; AOR, adjusted odd ratio; CI, confidence interval.

In the multivariable analysis, females (AOR = 0.61, 95% CI: 0.49–0.76, *p* < 0.001), postgraduate education (AOR = 0.48, 95% CI: 0.31–0.74, *p* = 0.001), and Phase-II (AOR = 0.62, 95% CI: 0.48–0.81, *p* < 0.001) remained associated with less vaccine acceptance. Participants with chronic diseases (AOR = 1.70, 95% CI: 1.23–2.33, *p* = 0.001) and who are generally motivated (AOR = 2.82, 95% CI: 2.29–3.47, *p* < 0.001) maintained higher vaccine acceptance. Other variables were not significant in the multivariable model, including those that were significant in the univariable analysis.

### Ranking predictors’ contribution to the phase-specific performance of high influenza awareness and vaccine acceptance through machine-learning models

**Figure 3.**
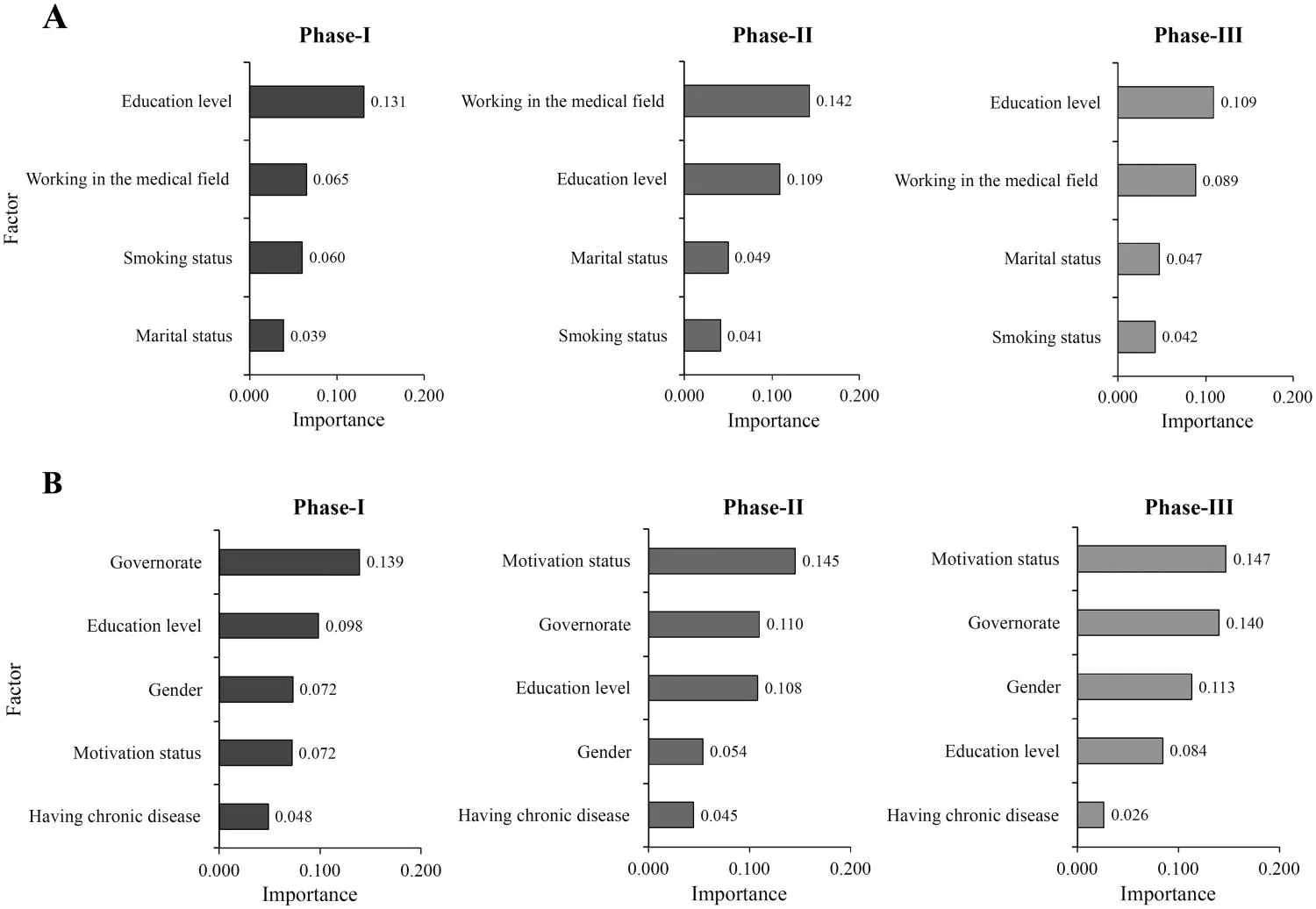
Contribution of predictors to the COVID-19 phase-specific models of high influenza awareness (A) and influenza vaccine acceptance (B).

The RF machine-learning model predicted “education level” and “working in the medical field” as major contributors to the “high influenza awareness” model across all phases (Figure 3A). On the other hand, the analysis for “willingness” showed that while participants’ location was an important contributor across all phases, the motivation inclination was the main contributor following the onset of the pandemic (i.e. in Phases II and III) (Figure 3B). These findings underscore the dynamic nature of feature contribution in predicting high awareness and willingness, indicating that while certain factors are universally significant, others may need to be weighed differently depending on the phase (Figure 3).

## Discussion

Since its outbreak, the coronavirus disease 2019 (COVID-19) has created an unprecedented global health crisis, affecting all aspects of life and causing health, economic, social, and educational impacts [1–3] with significant efforts being initiated to thwart the pandemic. Coinfection with COVID-19 and influenza has been associated with poor health outcomes [7,8]. Consequently, protection against influenza has become increasingly important during the COVID-19 pandemic. In fact, several countries have amended influenza vaccine mandates to reduce the potential co-circulation of influenza and COVID-19, both of which share similar clinical presentations and symptoms [26–28]. Despite increasing evidence of the benefits of the influenza vaccine and its favourable clinical outcomes during the COVID-19 pandemic, the vaccine remains excluded from the national immunization program in Yemen. Furthermore, the role of the COVID-19 outbreak in shaping influenza awareness and vaccination intentions [12,13] remains largely unknown in this war-torn country. In this study, we explore the temporal impact of the COVID-19 pandemic on public awareness of influenza and attitudes toward its vaccine and assess predictors of high influenza awareness and vaccine acceptance. Findings revealed that the COVID-19 pandemic led to an increase in public awareness of influenza but had a negative impact on the intention to receive the vaccine.

Assessment of participant awareness revealed a significant increase in their knowledge of influenza infection, how it is transmitted, and how it can be prevented. This outcome is not surprising, as both influenza and COVID-19 are contagious respiratory tract infections that share common transmission and prevention modes [7,8]. Consequently, promoting awareness of the transmission and prevention of one infection will likely enhance awareness of the other. Similar observations have been reported in other settings, where the pandemic increased public awareness of influenza [29]. However, the increase observed in Yemen is particularly notable given the country’s fragile healthcare system, the absence of a national seasonal influenza vaccination policy, the exclusion of influenza vaccine from the national immunization program, and the lack of previous influenza surveillance programs or awareness campaigns [17]. These findings suggest that major public health crises may substantially influence infection-related awareness even in settings facing considerable healthcare and socioeconomic challenges. Therefore, awareness campaigns during pandemics can be leveraged to raise awareness of other infectious diseases for which coinfection may be associated with poorer clinical outcomes.

Assessment of information sources participants relied on to learn about influenza showed that television was the primary source of influenza information in Phase-I, whereas physicians became the most frequently cited source in Phase-II. Several studies have shown that, following the onset of the COVID-19 pandemic, healthcare providers’ endorsement of the influenza vaccine became a significant factor in vaccine acceptance among participants [30,31]. Consequently, healthcare providers may capitalize on the COVID-19 crisis, and pandemics in general, to promote influenza vaccine acceptance, given its additional benefits during the COVID-19 outbreak. On the other hand, most participants in Phase-III sought information from multiple sources and/or explored alternative means to obtain information. These findings suggest a shift in the community’s information-seeking behaviour throughout the pandemic timeline, likely reflecting a tendency to expand the information sources consulted.

Interestingly, despite the significant increase in awareness in Phases II and III, there was a decrease in the intention to receive the influenza vaccine in those phases. This contrasts with several previous studies that report COVID-19 as a catalyst for increased willingness to receive the influenza vaccine [30,32,33]. Similarly, a systematic review and meta-analysis reported an overall increase in influenza vaccination intention following the onset of the COVID-19 pandemic across the 27 included studies, although the magnitude of this effect varied between studies and populations [34]. However, there was more consensus among participants in Phases II and III to be vaccinated if the vaccine was promoted by the government and/or physicians, demonstrated to be safe and effective, and/or offered at no cost by the government. These findings reflect a more reserved attitude toward influenza vaccine acceptance after the onset of the pandemic. The results suggest that the decreased intention to vaccinate could be multifactorial. Concerns about the safety and efficacy of the vaccine appear to be among the key factors, as participants were more willing to receive the vaccine when reassured of its safety and efficacy and when the vaccine was endorsed by experts (physicians) and authorities (government). Indeed, influenza vaccine hesitancy during the COVID-19 pandemic increased when concerns about the vaccine’s safety and efficacy outweighed the perceived risk of influenza and its complications [32,33]. The converse applies to those who accepted influenza vaccination [32,35]. Previous studies have also shown that influenza vaccine uptake increased among individuals who shared worries about COVID-19 infection [35–37]. In this regard, participants in our study with one or more chronic conditions (who, as a result, are at higher risk of complications from COVID-19 and influenza) were more likely to accept the influenza vaccine. Overall, the decreased intentions to receive the influenza vaccine following the onset of the COVID-19 pandemic in Yemen are concerning, given the country’s poor adherence to COVID-19 preventive measures [38]. To overcome these barriers, awareness campaigns should be implemented during pandemics through multimedia, healthcare providers, and social media platforms to emphasize influenza vaccination’s additional benefits during health crises.

The economic impact of the pandemic may have also negatively influenced influenza vaccine acceptance, as the vaccine is not offered free of charge in Yemen. This inference is supported by the more positive attitude toward accepting the vaccine in Phases II and III if it was provided at no cost. Further evidence of this economic burden affecting the decision to accept the vaccine can be seen in the greater willingness to receive the vaccine in Phase-III, compared to Phase-II, despite being lower than in Phase-I. This occurred after the pandemic had subsided, followed by a marginal improvement in the economic situation. The role of the economic burden of the pandemic in shaping the willingness to pay for the vaccine is expected in a low-income, developing country experiencing a decade-long, resource-depleting armed conflict. Thus, it is crucial to address misconceptions about the influenza vaccine and promote equitable access to vaccination during pandemics by offering it at no or minimal cost, and by considering its incorporation into the national immunization program in the country.

Education and working in the medical field both emerged as important predictors across the three phases in the high influenza awareness machine-learning model. On the other hand, governorate emerged as an important predictor across all phases in the influenza vaccine acceptance machine-learning model, although it is difficult to determine whether this governorate-based difference is related to differences in access to influenza vaccines across governorates, as the study was not designed to assess this variable. Motivation status was a strong predictor of vaccine acceptance in Phases II and III, but not in Phase-I, suggesting that factors other than motivation, such as educational level, are better predictors of vaccine acceptance in pre-pandemic and non-health-crisis situations.

In this context, many of the societal, healthcare, and economic changes that occurred in Yemen during the pandemic period, including changes in the public health situation, healthcare utilization, and social behaviours, arose as direct or indirect consequences of the COVID-19 pandemic. Such changes may have influenced certain characteristics of subsets of participants and, consequently, contributed to the temporal changes observed in influenza-related knowledge and attitudes toward influenza vaccination.

The limitations of this study highlight opportunities for improvement in future research. Although the study sampled populations from different locations and cities, the sampling method remains convenience sampling. Implementing random sampling across the population would have been logistically difficult due to the unique challenges associated with the country and the timing of data collection. These challenges include the ongoing armed conflict, political disputes, and the outbreak of the COVID-19 pandemic, along with its associated restrictions. Nonetheless, the study provides valuable insights into the circumstances arising from the overlap of pandemics with other viral infections in a country facing unique challenges and paves the way for future research to further explore these circumstances.

## Conclusion

The impact of pandemics on public awareness and attitudes toward vaccines has become more evident following the COVID-19 outbreak. While the increased awareness of influenza is a positive finding, the decreased acceptance of the vaccine following the onset of the pandemic is both discouraging and alarming, underscoring a negative health-seeking behaviour by the community. Evidently, promoting positive attitudes and practices toward the influenza vaccine may encourage acceptance of other vaccines and lead to higher vaccination rates during future pandemics and against diseases with significant public health implications [34]. Thus, the changes in awareness and attitudes observed in this study may serve as a proxy for vaccination uptake behaviour in the country across the pandemic timeline. Furthermore, these findings can inform decision-making authorities, aiding in the effective design and implementation of awareness and vaccination campaigns tailored to address the unique challenges faced by fragile nations during pandemics.

## Data Availability

The data supporting the findings of this study are available from the corresponding author upon reasonable request.
